# Tempering expectations: considerations on the current state of stem cells therapy for autism treatment

**DOI:** 10.3389/fpsyt.2023.1287879

**Published:** 2023-10-03

**Authors:** Antonio Narzisi, Alycia Halladay, Gabriele Masi, Gaia Novarino, Catherine Lord

**Affiliations:** ^1^Department of Child Psychiatry and Psychopharmacology, IRCCS Stella Maris Foundation, Calambrone, Pisa, Italy; ^2^Autism Science Foundation, New York, NY, United States; ^3^Department of Pharmacology and Toxicology, Rutgers University, Piscataway, NJ, United States; ^4^Institute of Science and Technology Austria, Klosterneuburg, Austria; ^5^Department of Psychiatry and Human Development and Psychology, University of California, Los Angeles, Los Angeles, CA, United States

**Keywords:** autism, stem cells, intervention, treatment, children, evidence-based

## Introduction

Autism spectrum disorder (ASD) is a genetically and phenotypically heterogeneous disorder (1, 2) and it affects 1 out of 36 children (3). Due to its heterogeneity, the causes of ASD are still poorly understood and scientific research is now focused on the early identification of bio-behavioral markers to anticipate the age of diagnosis (4). Making an early diagnosis has positive implications in terms of implementation of timely evidence-based interventions and, consequently, better outcomes (5). In the complex arena of interventions for ASD, some of them are evidence-based, while others (a) are proposed without scientific basis (6), or (b) they have not yet completed the necessary steps to move from basic research to large-scale clinical application but are transferred to clinical practice. Regarding option *b*, in recent years, we have witnessed a worrying increase in institutes that proposing to families to treat ASD with stem cells from various sources, including those obtained from cord blood (7). The alarming aspect of this potential therapeutic proposal is the promise of significant clinical improvements in children who undergo this treatment. These institutes, which are often located in countries with low medical standards, are not proposing a research trial but the use of stem cells as a therapeutic option already validated by basic research. However, to date, can we say that the use of stem cells is an evidence-based treatment? The answer is no, and we will try in the following lines to explain the reasons for this negative answer.

## Main steps of biomedical research

Biomedical research is divided into several steps, including basic research and translational research. Basic research focuses on understanding biological and physiological mechanisms, while translational research is geared toward transferring the knowledge gained in basic research into practical applications, such as new therapies, medical devices, or prevention strategies (8). The main steps from basic to translational research include (a) Identification of scientific discovery (basic research may lead to the discovery of new biological mechanisms or to an understanding of how diseases develop) (9); (b) Development of a therapeutic strategy [the scientific discovery can be used to develop a therapeutic strategy, such as a new drug molecule, gene therapy or medical device (10)]; (c) *In vitro* and *in vivo* evaluation [the new therapeutic strategy is tested *in vitro*/in the laboratory and *in vivo* (on animals) to assess its efficacy and safety] (11); (d) Clinical trial (if the new therapeutic strategy shows promise *in vitro* and *in vivo* tests, it can be submitted to clinical trials in human volunteers) (12); (e) Regulatory authorization (after successfully passing the clinical trial phase, the new therapeutic strategy can be authorized by regulatory agencies to be marketed and used as a medical treatment) (13); (f) Clinical application (the new therapeutic strategy can be used in clinical practice to treat patients suffering from specific diseases) (12); (g) Monitoring and improvement (after clinical application, the new therapeutic strategy is monitored to assess its long term efficacy and safety and to make any improvements) (12). It is possible to consider these main steps of biomedical research the scientific background from which the Empirically Supported Treatments (ESTs) emerge. The ESTs are treatments that have demonstrated their effectiveness through rigorous scientific research and controlled studies (14, 15). This means they are supported by empirical data showing that the treatment is superior to a placebo or alternative treatments for a specific disorder (14). By the way, to be classified as an EST, a treatment must have undergone numerous randomized controlled clinical studies that consistently demonstrate its efficacy (15). These studies must be conducted using rigorous scientific methods and published in peer-reviewed journals (14, 16). The ESTs are often recommended as the first-line treatments for specific disorders (15). This implies that when a clinician must choose a treatment for a patient, they should first consider ESTs, as they have a solid empirical basis of support. While ESTs provide important guidance in treatment selection, it does not mean that all patients should receive the same treatment. Clinicians should also consider individual patient needs and tailor treatments accordingly (17).

## Expert opinion

To date, research on the use of stem cells for ASD is at the clinical trials stage (18–29) and the results, although potentially encouraging in terms of safety (30, 31), are not yet sufficient to allow their clinical application. Safety in fact should be established by open-labeled phase I/II trials, which are very few at present (32). Most published studies do not have a standardized and shared protocol of evaluation (18, 20, 33–35); do not describe a standardized method of treatment (22, 24, 25, 27, 34, 36); and have small sample size (18, 33, 34). There are no robust and significant clinical differences for any endpoints (32). For example, among the methodologically well-conducted studies, the Chez research (20) that employed a placebo controlled, cross-over design and involved 29 children between the ages of 2.4 and 6.8 years reported no significant changes in any of the behavioral tests (including vocabulary tests, cognitive assessments, socialization, and communication evaluations) throughout the 49 weeks of the study (32). In contrast, the study by Dawson et al. (19), which was an open-label study on 25 children of similar age showed significant improvements in socialization, communication, adaptive behaviors, and eye tracking. These effects were observed at 6 months and remained stable over the 12-month study period. However, as pointed out by Price (32) the main difference between the two studies is the use of a placebo- controlled design vs. an open-label design. It is also noteworthy that the improvements seen in the Dawson study align with those observed in control patients from a Swedish cohort of similar age (37), suggesting they may be attributed to the natural course of the disorder. To date, these relatively large and well-designed studies provide little support for the therapeutic use of cord blood mononuclear cells (CBMCs) in ASD. Furthermore, there are no robust data on the mid- and long- term effects of treatment (18, 20, 32–35) and, as mentioned above, safety and feasibility of stem cell administration in children with ASD have not been well-established (32, 36). There is little scientific rationale for why stem cells would be effective. Indeed, (a) the ASD is not a neurodegenerative disorder (one of the primary reasons for the limited scientific rationale regarding the efficacy of stem cells in ASD treatment is that ASD is not a neurodegenerative disease. Neurodegenerative diseases involve the progressive degeneration of specific brain cells, such as in conditions like Alzheimer's or Parkinson's disease. In these conditions, stem cells could be used to replace damaged or dead cells. ASD, on the other hand, involves the complex interplay of many genes and different brain areas. There is no specific type of cell to replace in the treatment of ASD); (b) there is wide individual variation in ASD (another challenge in treating ASD with stem cells is the considerable variation among autistic individuals. Each person with ASD has a unique profile of symptoms, making it difficult to identify a uniform therapeutic approach that works for everyone. This means that even if an effective stem cell therapy were developed, it might not work for all autistic individuals); (c) the critical developmental period is unknown (some stem cell-based treatments are successful when administered during a critical period of development. However, the exact timing when a stem cell intervention might be effective for ASD remains unknown. The ASD is a complex condition that begins to develop during pregnancy and continues in the early years of life. Determining the optimal timing to administer stem cell treatment is challenging); (d) there is limited clinical evidence (most stem cell-based therapies for ASD are still in the preclinical or early research phase. There is limited strong clinical evidence demonstrating the efficacy and safety of these therapies in the context of ASD. This lack of clinical data is one of the primary reasons why stem cell treatment for ASD is still considered experimental). Moreover, in absence of myeloablative pre-conditioning, little or no engraftment would be expected from the transplantation of autologous or allogeneic umbilical cord blood cells. As a rationale for the use of stem cells in autism therapy, some studies have reported the so-called immune brain axis (38), although it is correct to point out that published studies to date have not done patient stratification to test the effect of the intervention on patients with ASD + immune dysfunction (39, 40). In addition, the ability of cord blood cells to form the neural cells whose function may be altered in ASD may depend on many factors such as for example, the type of stem cells used the method of administration and the cellular environment (41). To date, although we have some interesting findings that have indicated positive effects in the context of neurological conditions such as releasing growth factors or supporting regeneration and repair of damaged brain tissue (42) less is known about the molecular action mechanisms of stem cells (43). In addition, as different forms of ASD are associated with alterations in distinct cell types, a one size-fits-all approach may not be just not advisable but even harmful. As a medical product that could cause serious harm to individuals receiving such transplants, regulatory agencies should continue and pursue monitoring the marketing and commercialization of stem cell treatments in the absence of solid evidence. The transition to the clinical application of stem cell transplants without having respected the previous steps and, therefore, without having guidelines and reliable and robust risk information could harm children and their families. Firstly, there is inadequate clinical evidence to support expanded access to this treatment for intermediate-size patient populations according to US Food and Drug Administration guidelines (44), ISSCR guidelines for Stem Cell Research and Clinical Translation (45) and Standards for Human Stem Cell Use in Research (46). There is a paucity of registered clinical trials for the use of umbilical cord blood-derived products for the treatment of ASD. Secondly, it is unethical and premature to allow such treatments to be marketed to families. Thirdly families should understand that, in certain cases, conflict of interest might involve University, the Commercial Entity, and the Researcher. While the use of patient- derived (induced pluripotent) stem cells in basic and applied translational research is based on profound scientific hypotheses and evidence, with this paper, we call on stronger regulatory control over stem cell treatments derived from umbilical cord blood by agencies that are designed to protect the health and wellbeing of some of the most vulnerable communities. Our brief paper is intended to promote high-level research on the use of stem cells, not to banish it. However, the transition from research results, albeit encouraging, to large-scale clinical application requires repeated scientific evidence on large samples and more large placebo controlled double-blind trials and exhaustive investigations which we do not yet have (47).

In agreement with Price (32) we believe that to continue in this therapeutic direction, preclinical studies should be conducted with the aim of improving patient stratification, biomarkers, the defined mode of action, and the preparation and identification of the therapeutic cells themselves.

## Author contributions

AN: Conceptualization, Supervision, Writing—original draft, Writing—review and editing. AH: Supervision, Writing—original draft, Writing—review and editing. GM: Supervision, Writing—review and editing. GN: Supervision, Writing—review and editing. CL: Supervision, Writing—review and editing.
